# A piece of the ‘chemobrain’ puzzle: pNF-H

**DOI:** 10.18632/aging.100751

**Published:** 2015-05-20

**Authors:** Akina Natori, Toru Ogata, Hideko Yamauchi

**Affiliations:** Department of Breast Surgery, St. Luke's International Hospital, Tokyo, Japan

Although chemotherapy can offer long-term survival for patients with cancer, recent reports [1] have revealed that it may induce cognitive dysfunction, such as deficits in attention, concentration, executive function, and processing speed. Whereas such chemotherapy-induced cognitive impairment (CICI) is usually less severe and most often transient, it sometimes impairs activities of daily living and quality of life to the point of debilitation. While it has been recognized as a clinically significant issue in patients, little is known about CICI, particularly in older cancer patients to whom chemotherapy has become more commonly proposed. CICI has been particularly studied among middle-aged women undergoing chemotherapy for breast cancer, while only a small number of studies have focused on older patients. Many of the candidate mechanisms for breast cancer chemotherapy-related brain injury overlap significantly with those involved in aging [2]. Therefore, CICI can be an interesting model for examining the interactions in disease, aging, and neurodegeneration. Here, we introduce some proposed mechanisms which CICI has in common with other types of dementia, and discuss the difficulties in evaluation of cognitive function in aged cancer patients.

One proposed mechanism of CICI is direct neurotoxicity by chemotherapy itself [1]. Magnetic resonance imaging studies have demonstrated lower integrity of cerebral white matter (the location of myelinated axons) rather than gray matter (the location of neuronal cell bodies) in patients with CICI in comparison with healthy subjects [3]. However, these findings do not directly indicate whether the decreased integrity of the cerebral white matter is caused by damage to axons themselves, which are the main components of white matter, or by Wallerian axonal degeneration following neuronal damage. Various tissues in the central nervous system (CNS), including neurons, axons, and glia, release several lines of proteins into the cerebral spinal fluid (CSF) and/or peripheral blood flow when damaged. Some of these proteins in the CSF and/or blood have been explored as objective biomarkers of the severity of neuronal damage.

Recently, we evaluated in a cross-sectional study the serum level of the phosphorylated form of the high-molecular-weight neurofilament subunit (pNF-H), a major structural protein in central and peripheral axons, in breast cancer patients undergoing adjuvant chemotherapy [4]. The results showed that the serum pNF-H level in patients increased in a cumulative, dose-dependent manner. Axonal damage in the CNS can be cumulatively caused by chemotherapy, which might eventually lead to CICI. Surprisingly, increased pNF-H levels in CSF were also observed in a previous study [5] in patients with Alzheimer disease when compared with age-matched controls and patients with non-Alzheimer disease-related neurological disorders and vascular dementia. Both studies suggest that the increased level of pNF-H could be used as a marker for neuro-degenerative disorders. Another study showed that the pNF-H level is associated with the severity of spinal cord injury, and may have adequate sensitivity to serve as a biomarker of treatment efficacy in patients with such injury [6]. It might be useful, therefore, to investigate severity indices of CICI and neural toxicity of chemotherapy on the CNS using pNF-H as a surrogate marker, rather than subjective cognitive test batteries.

Another explanatory mechanism of CICI is the patient's genetic status, such as apolipoprotein E (APOE), which is also associated with cognitive decline related to Alzheimer's disease and aging [7]. APOE status influences the clearance of amyloid-beta, which is deposited in the brain with aging and is a key element in the pathology of age-related cognitive decline and neurodegeneration. Chemotherapy itself may increase amyloid-beta accumulation through a rise in inflammation and oxidative stress, altered glucose metabolism, and other factors. A previous study showed that the epsilon-4 allele of APOE may be a potential genetic marker for increased vulnerability to chemotherapy-induced cognitive decline [7]. Further studies combining APOE status and amyloid-beta are needed to examine the interactions between aging, amyloid-beta accumulation, genetic risk, and chemotherapy.

The evaluation of CICI is also challenging. CICI is moderate and often transient, so many cognitive screening tests used in oncogeriatry, such as the Comprehensive Geriatric Assessment in Oncology and Mini Mental State Examination, are not sensitive enough to detect CICI in both young and aged cancer patients. Therefore it may be promising to use biomarkers such as pNF-H as prognostic markers, rather than subjective cognitive tests, to detect CICI, or to employ them as predictive markers to distinguish which patients would develop CICI before symptoms are recognized.

In summary, although the effects and mechanisms of chemotherapy on the cognitive functions remain unclear, some may be similar to the neurodegenerative disorders associated with aging. Further studies of biomarkers, such as pNF-H, might help us to understand the mechanisms of both CICI and brain aging, measure the severity of CICI, and predict who may develop CICI.
